# Supplementary material for: Seminoma and Embryonal Carcinoma Footprints Identified by Analysis of Integrated Genome-Wide Epigenetic and Expression Profiles of Germ Cell Cancer Cell Lines
Source: PLoS One. 2014 Jun 2;9(6):e98330. doi: 10.1371/journal.pone.0098330 (PMC4041891; doi:10.1371/journal.pone.0098330)
Supplement: File S1 — ZIP file containing DMRforPairs output for significant regions. Please start from the html files. (ZIP) [file pone.0098330.s008.zip › figures/14779.pdf]

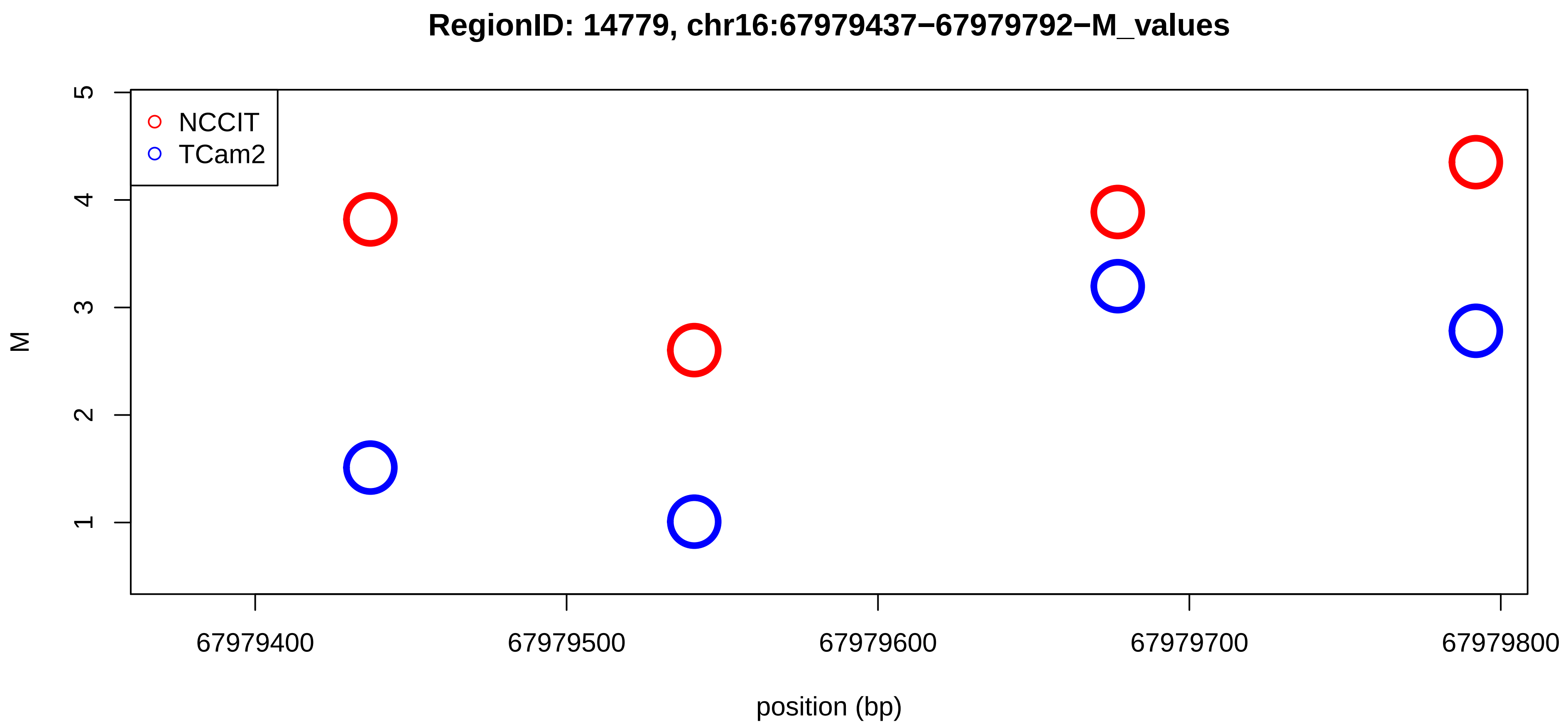
RegionID: 14779, chr16:67979437−67979792−M_values
NCCIT
TCam2
M
1
2
3
4
5
67979400
67979500
67979600
67979700
67979800
position (bp)

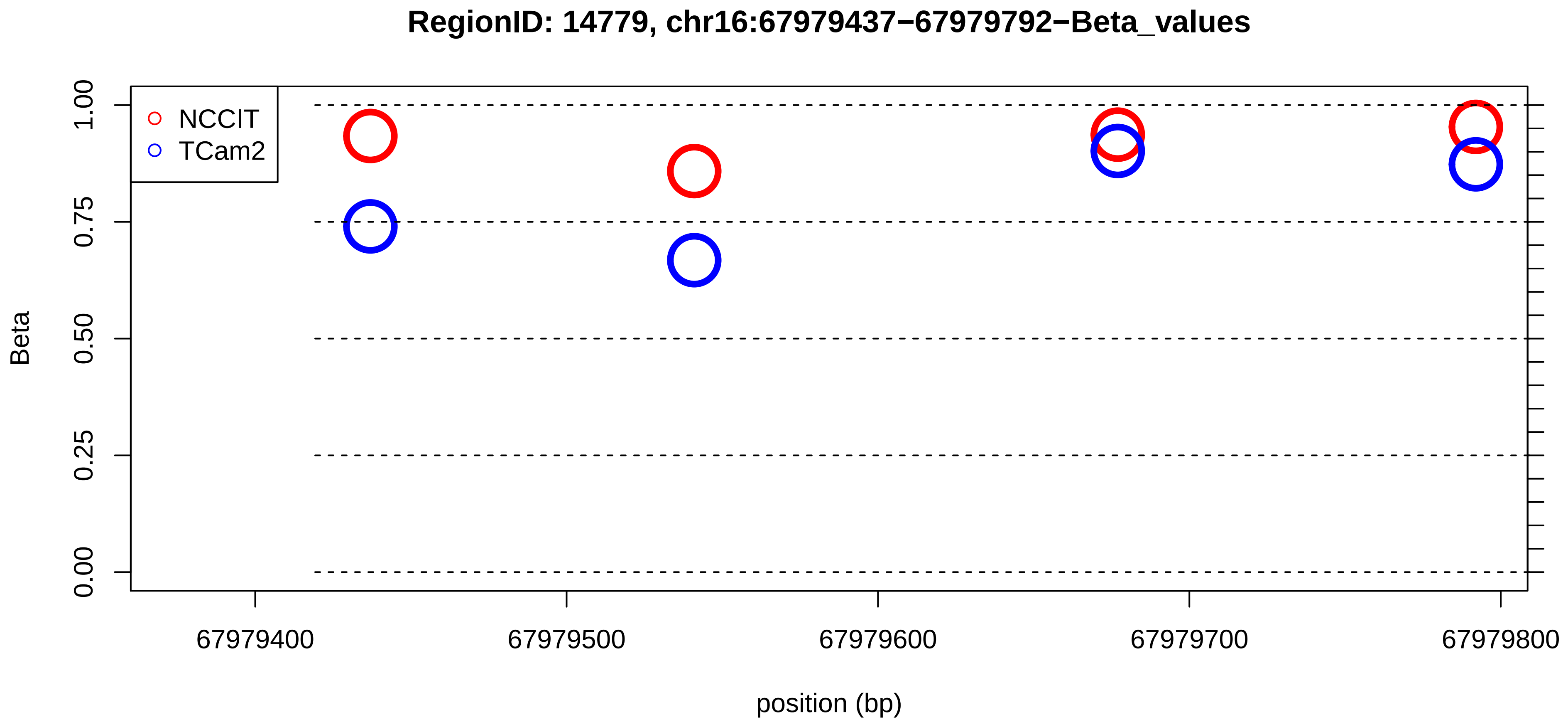
RegionID: 14779, chr16:67979437−67979792−Beta_values
NCCIT
TCam2
Beta
0.00
0.25
0.50
0.75
1.00
67979400
67979500
67979600
67979700
67979800
position (bp)
